# AlphaFold2-based prediction of the co-condensation propensity of proteins

**DOI:** 10.1073/pnas.2315005121

**Published:** 2024-08-12

**Authors:** Shengyu Zhang, Christine M. Lim, Martina Occhetta, Michele Vendruscolo

**Affiliations:** ^a^Centre for Misfolding Diseases, Yusuf Hamied Department of Chemistry, University of Cambridge, Cambridge CB2 1EW, United Kingdom

**Keywords:** Alzheimer’s disease, Parkinson’s disease, liquid–liquid phase separation, supersaturation, protein misfolding

## Abstract

The process of protein phase separation into liquid condensates has been implicated in the formation of membraneless organelles (MLOs), which selectively concentrate biomolecules to perform essential cellular functions. Although the importance of this process in health and disease is increasingly recognized, the experimental identification of proteins forming MLOs remains a complex challenge. In this study, we addressed this problem by harnessing the power of AlphaFold2 to perform computational predictions of the conformational properties of proteins from their amino acid sequences. We thus developed the CoDropleT (co-condensation into droplet transformer) method of predicting the propensity of co-condensation of protein pairs. The method was trained by combining experimental datasets of co-condensing proteins from the CD-CODE database with curated negative datasets of non-co-condensing proteins. To illustrate the performance of the method, we applied it to estimate the propensity of proteins to co-condense into MLOs. Our results suggest that CoDropleT could facilitate functional and therapeutic studies on protein condensation by predicting the composition of protein condensates.

## Significance

The phenomenon of protein condensation has been associated with a variety of cellular functions and implicated in a wide range of human diseases. By developing CoDropleT, a predictive model incorporating conformational properties of proteins derived from AlphaFold2, this study introduces a tool for the prediction of proteins that make up protein condensates. This tool could be used for accelerating the finding of proteins involved in protein phase separation and for elucidating the still elusive mechanisms governing protein co-condensation. We also expect CoDropleT to contribute to future explorations of the therapeutic opportunities offered by the modulation of pathological processes generated by the dysregulation of protein condensates.

Eukaryotic cells contain two major types of organelles that contribute to the spatio-temporal regulation of biological processes, depending on whether or not they are enclosed by lipid membranes (1). Membraneless organelles (MLOs) are typically composed of proteins and protein–RNA complexes, and retain relatively stable structures that can compartmentalize and concentrate specific sets of molecules (1–6). Examples of MLOs include nuclear structures, such as Cajal bodies, nucleoli, and nuclear speckles, as well as cytoplasmic structures, such as signaling complexes, postsynaptic densities, P-bodies, stress granules (SGs), and germ-line granules (1–6). MLOs have been implicated in a variety of cellular processes (1–8), including stress response (6, 9), regulation of gene expression (10, 11), and the control of signal transduction (12–14).

Recent studies have reported that MLOs exhibit biophysical properties characteristic of liquids, suggesting that they could be considered as liquid-like condensates (2–16). For instance, germ-line P granules in *Caenorhabditis elegans* adopt spherical shapes, coalesce into increasingly larger droplets when they come into contact (2, 17), and display the ability to wet intracellular surfaces such as the nuclear envelope (3). Furthermore, the molecules within these condensates are in dynamic interchange with the surrounding nucleoplasm and cytoplasm (14, 17). These observations collectively suggest that MLOs could be liquid condensates that form via a biologically regulated protein phase separation (PPS) process, also referred to as condensation or droplet formation (2–17).

Intriguingly, aberrant PPS processes have been associated with several human pathologies, including neurodegenerative diseases, by facilitating the formation of aberrant solid-like aggregates (18–20). A common feature among proteins associated with these diseases is their ability to undergo PPS, forming dynamic condensates that play important roles in physiological cellular processes. Thus, understanding the mechanisms governing PPS and the transitions between protein states is vital for the development of therapeutic strategies for such conditions (18–20).

Given the great technical challenges in the quantitative description of the phase separation process in the cell by experimental methods (21, 22), computational methods can facilitate the identification of proteins with a high propensity to undergo PPS (23–26). First-generation methods for the prediction of the PPS propensity mainly rely on the relationship between conserved sequence properties and phase separation (24). Noteworthy among them are PLAAC (27), FuzDrop (28, 29), LARKS (30), DDX4-like (31), and several others (32–35), each employing different approaches to predict phase separation propensity based on sequence attributes. More recently, the attention is shifting towards machine learning models, which can capture complex, nonlinear relationships between variables, making them well-suited for tasks like PPS prediction, which involves an understanding of the complex interactions between amino acids and their environment. These methods typically involve training a model on large datasets of proteins with known phase separation propensities. A model learns to recognize patterns in the data that are associated with phase separation and can then use these patterns to predict PPS in proteins not present in the training set. Random forests and gradient boosting machines are used by PSAP (23) and dSCOPE (36), and PSPredictor (37) and PICNIC (38), respectively. The advent of neural networks and language models has further enriched the landscape of phase separation prediction, with tools such as DeePhase (39), BERTIG (40), LLPhyScore (41), and PhaSePred (42).

However, despite recent advances (38, 42–44), a significant gap largely remains—the ability to accurately predict the co-condensation propensity of proteins. To address this problem, we report CoDropleT, a method of predicting protein co-condensation by integrating information regarding the conformational properties of proteins, using the structural representations generated by AlphaFold2 (45). Since PPS depends on the conformational properties of proteins, our inductive bias is that this approach should help enhance the model performance, in particular considering the possibility that AlphaFold2 could generate accurate predictions also for disordered proteins (46). Our expectation is that the use of AlphaFold2 should lead to high-quality predictions, since transformers, with their self-attention mechanisms, can handle long-range dependencies in the sequence data. These long-range dependencies are crucial in proteins, as their functions are often determined by interactions between residues far apart in the sequences but close together in the folded structures. Transformers are also designed to handle sequences of varying lengths, allowing them to deal with proteins of any size. Furthermore, unlike recurrent neural networks, which process the input sequence one element at a time, transformers can process all elements of the input sequence in parallel. This makes them computationally efficient and allows for faster training, which can be particularly useful when dealing with large protein datasets.

The results from the proof-of-concept analysis reported in this work indicate that a transformed-based approach is well-equipped to handle the complexities and challenges associated with predicting protein cocondensation in PPS.

## Results

### Overview of the CoDropleT Architecture and Algorithm.

**Fig. 1.**
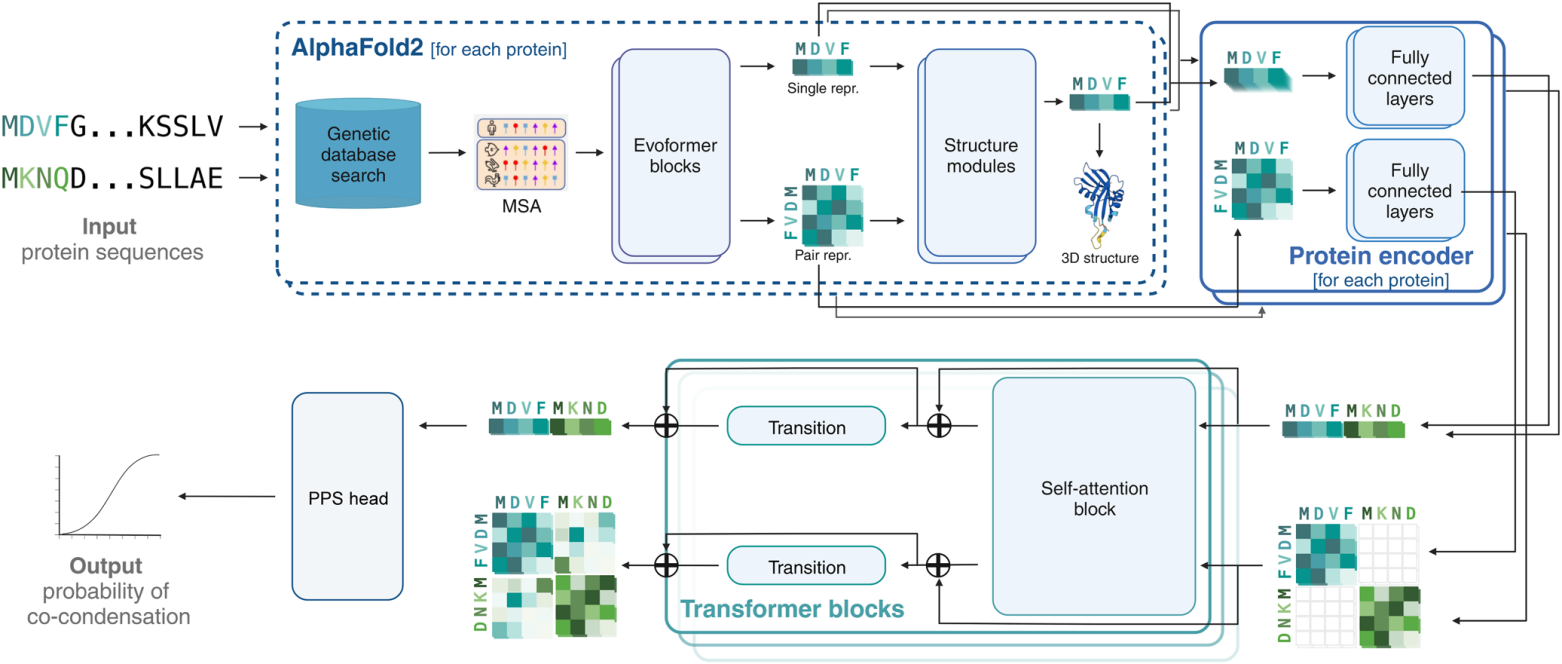
Schematic representation of the computational workflow for CoDropleT. The process begins with input protein sequences that are subjected to a genetic database search to create a multiple sequence alignment (MSA). Each protein sequence is then independently processed by the AlphaFold2 system (45), which utilizes Evoformer blocks to generate single and pair representations of the protein sequences. A protein encoder further processes the sequences through fully connected layers. The combined output from the protein encoder of each protein is then fed into transformer blocks with self-attention mechanisms, allowing for the capture of complex sequence and structural features. Finally, a PPS head analyses the features to predict the output probability of co-condensation between the protein pairs.

CoDropleT was constructed using the Haiku framework, which was developed by DeepMind to design neural networks within JAX (47) and takes as input pairs of protein sequences (*Materials and Methods* and Fig. 1). This input is fed into the pretrained AlphaFold2 model (45), resulting in two distinct outputs: a single representation and a pair representation. The representation of each protein is separately generated with the same encoder. The two representations are then concatenated into a single graph. We also add a relative positional encoding to the pair representation, which is a binary value used to distinguish whether a residue pair is from the same chain or from a different chain. Once the representations have been encoded, information can be exchanged between the two within the transformer blocks, which include a self-attention block followed by a transition layer for nonlinear transformation. The data are fed through eight layers of transformer modules to obtain final representations of each residue, which are then pooled to predict the propensity for coseparation (Fig. 1).

### Assessment of the Quality of the CoDropleT Predictions.

**Fig. 2.**
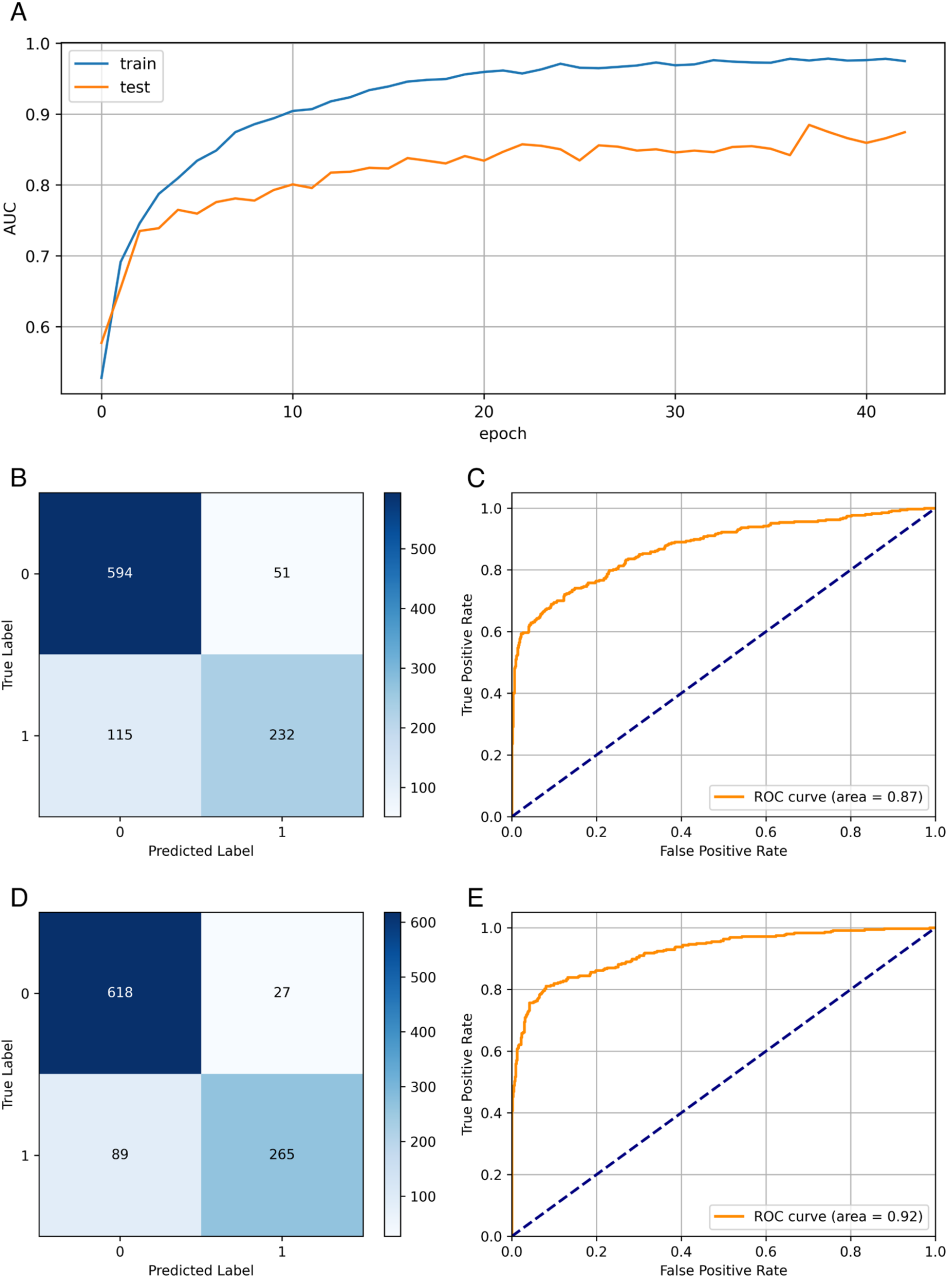
CoDropleT evaluation metrics. CoDropleT was trained on a PPS-positive dataset built by forming protein pairs using the CD-CODE condensates with *n* = 50, and a PPS-negative dataset built by pairing proteins that undergo phase separation, but not together, with *m* = 5. This model was trained and tested using the refined dataset, which we ensured did not contain overlaps between condensates present in the training and test sets. (*A*) AUC against the number of training epochs. (*B*–*E*) Confusion matrices (*B* and *D*) and ROC curves (*C* and *E*), before and after incorporating additional data of protein pairs that form by themselves.

CoDropleT was trained on the PPS-positive and PPS-negative datasets (*Materials and Methods*). We considered the possible presence of overlaps between the training and test sets, as such overlap might lead to an artificial increase in the confidence in the predictions. For instance, if the training set contains the pairs of proteins (a,b) and (a,c) from a condensate containing the proteins a, b, and c, the pair (b,c) should not appear in the test set. We thus split the training and test set based on the condensates, ensuring that positive protein pairs in the training and test sets are from different condensates. CoDropleT achieved an accuracy of 0.833, precision of 0.669, recall of 0.820, F1 score of 0.737, area under the curve (AUC) of 0.874, and Matthews correlation coefficient (MCC) of 0.623 (Fig. 2). To address a potential bias arising from having trained CoDropleT on only condensate-localizing proteins, we further expanded the dataset to include protein pairs that self-condensate (*Materials and Methods*). Upon further training (20 epochs) on the expanded dataset, our final model showed improved performance on the same test set, with an accuracy of 0.884, precision of 0.749, recall of 0.908, F1 score of 0.820, AUC of 0.923, and MCC of 0.743.

### Prediction of Protein Co-condensation in MLOs.

**Fig. 3.**
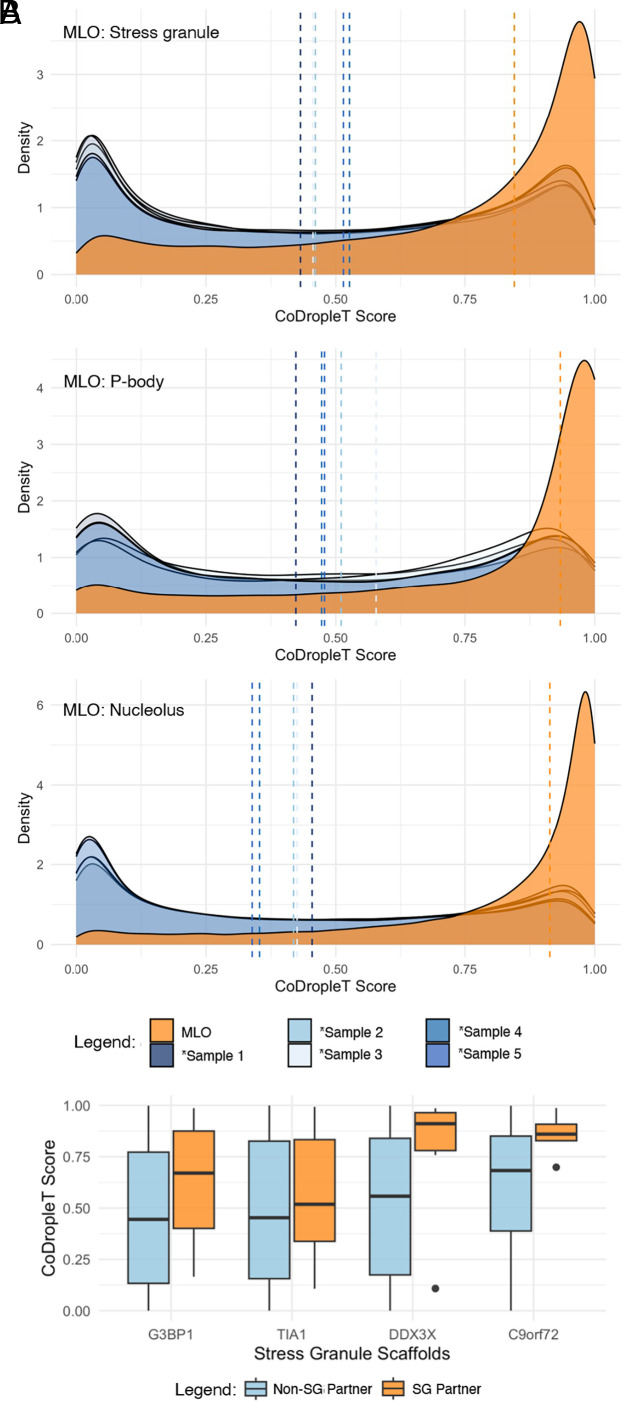
Pairwise CoDropleT scores for MLO proteins against random set of proteins of the same size. (*A*) Distribution of CoDropleT scores (orange) for protein pairs localizing within nucleoli, P-bodies, and SGs. For comparison, we show five distributions of CoDropleT scores (*blue) for protein pairs localizing within five randomly sampled protein sets of the corresponding size. Dashed lines represent the median values of the distributions. (*B*) Average pairwise CoDropleT co-condensation propensity scores of four protein scaffolds with a set of high-confidence SG protein partners (orange) and non-SG protein partners (*blue). Protein scaffolds tend to have higher co-condensation scores with the SG protein set as compared to non-SG proteins.

An important application of methods of predicting co-condensating proteins is the composition of MLOs. As proof-of-concept to estimate the ability of CoDropleT to predict protein co-condensation propensity, we performed an analysis of proteins in three well-characterized MLOs: SGs, P-bodies, and nucleoli. We compared the predicted pairwise co-condensation propensities of proteins reported to localize within each MLO against five random samples of proteins of the same size as the tested MLO (*Materials and Methods*). Our results reveal that CoDropleT is consistently able to predict relevant cocondensing protein pairs across all three MLOs (Fig. 3*A*).

### CoDropleT Differentiates Relevant Sets of Potential Client Proteins.

Next, we asked whether or not CoDropleT can predict client proteins in MLOs of interest. In this section, we study four known protein scaffolds of SGs: G3BP1, TIA1, DDX3X, and C9orf72 (48). Since these protein scaffolds are essential for modulating SG formation, they could be expected to exhibit a higher co-condensation propensity with the set of proteins that localize to SGs as compared to other proteins. Given this assumption, we compared the CoDropleT co-condensation propensity scores of each of the four scaffolds with high-confidence SG components (*Materials and Methods*) against their co-condensation propensities with non-SG components. We found that the scaffolds tend to have higher co-condensation scores with the SG protein set as compared to non-SG proteins (Fig. 3*B*). These results suggest that CoDropleT may help the identification of client proteins that co-condensate with protein scaffolds of interest.

### Prediction of Co-condensating Proteins in the Cytosolic Human Proteome.

**Fig. 4.**
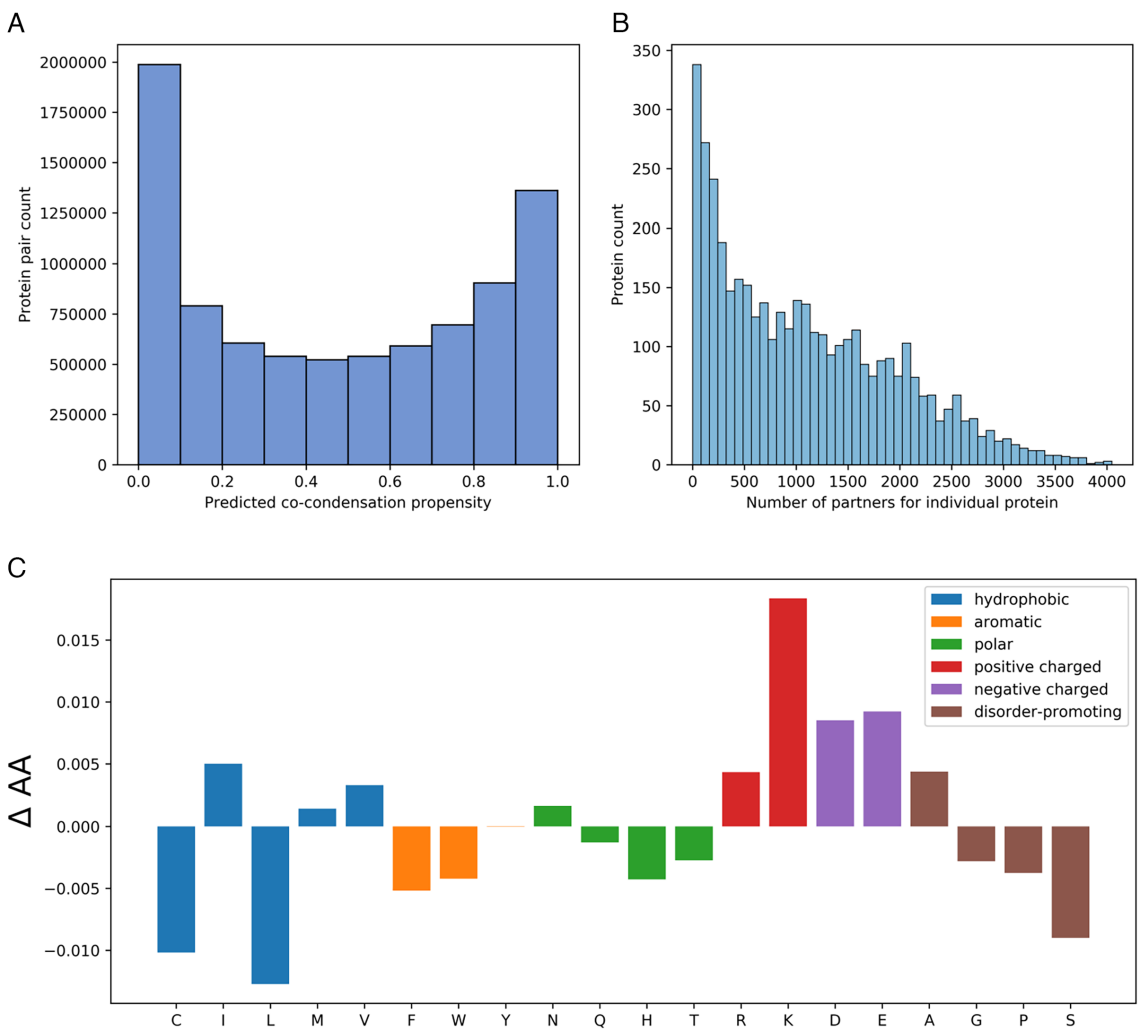
Predictions of co-condensation propensity of cytosolic human protein pairs. (*A*) Predicted co-condensation propensities for protein pairs, with the *x*-axis denoting the propensity score ranging from 0.0 to 1.0 and the y-axis representing the count of protein pairs for each propensity score bin. (*B*) Number of partners for individual proteins, where a partner is characterized by having a predicted co-condensation propensity score above 0.8. The *x*-axis specifies the number of partners, and the *y*-axis displays the count of proteins with that number of partners. (*C*) Differences in amino acid composition between proteins with a large number of predicted possible partners (>1,908, n = 827) and those with fewer predicted possible partners (<233, n = 827). Amino acids have been categorized as hydrophobic (blue), aromatic (orange), hydrophilic (green), positively charged (red), negatively charged (purple), and disorder-promoting (brown). The SE and significance of the differences are shown in *SI Appendix*, Table S1.

To explore the applicability of CoDropleT to perform proteome-wide predictions, we used it to estimate the co-condensation propensities of all possible protein pairs within the cytosolic human proteome. Our analysis indicated that approximately 25% of the cytosolic human protein pairs are predicted to have a co-condensation score above 0.8 (Fig. 4*A* and Dataset S1). When examining the number of partners for individual proteins, we observed that a few proteins have many partners (Fig. 4*B* and Dataset S2). Next, we analyzed the difference in amino acid composition between proteins with many predicted possible co-condensation partners (>1,908, n = 827) and proteins with few predicted possible co-condensation partners (<233, n = 827) (Fig. 4*C*). Our results revealed that proteins with a high number of interacting partners tend to have an increased fraction of charged residues (arginine [R], lysine [K], aspartic acid [D], and glutamic acid [E]). Simultaneously, these proteins show a decreased fraction of residues that promote disorder, specifically glycine [G], proline [P], and serine [S], but not alanine [A]. Additionally, they are characterized by a lower occurrence of some hydrophobic residues, notably cysteine [C] and leucine [L].

## Discussion

We have reported CoDropletT, a method of predicting the propensity of proteins for co-condensation. CoDropleT is aimed at bridging the gap between in vitro studies of phase separation of individual proteins and in vivo studies of MLO composition and function. The ability of CoDropletT to predict co-condensating proteins could help advance our ability to study PPS in complex environments.

In this discussion, we highlight the complexity in predicting PPS and emphasize the need for further model refinement and validation. Although, as we have shown, CoDropleT shows promising results in capturing protein compositions of MLOs and scaffold-client relations, there is considerable scope to improve model performance in future work. One could achieve this by refining the datasets used for training and validation, possibly leading to improvements in performance. The absence of large, standardized datasets containing proteins with clear experimental validation limits the data available for training, in turn raising the risk of overfitting. In addition, building PPS-negative datasets remains a complicated task due to the lack of publicly available resources that report non-condensate forming proteins and highlights the need for robust carefully curated datasets. Future efforts will need to focus on the synergy between computational and experimental approach to develop a comprehensive and accurate PPS negative dataset, which is critical for training and validating predictive models. Furthermore, CoDropleT is currently largely based on the intrinsic properties of protein sequences and it does not consider extrinsic factors that account for the fact that protein condensates form under specific conditions. The integration of features such as interactions with nucleic acids into the predictions can provide a more detailed picture of how PPS occurs within the cellular milieu. By incorporating these additional variables, predictive models may become better capable of capturing the multifactorial nature of phase separation, thus offering predictions that better reflect the biological complexity of this process. Finally, experimental validation of our findings would also be a pivotal next step to confirm the predictions of our models.

In conclusion, CoDropleT is a deep learning method of predicting the co-condensation of proteins into liquid condensates. This approach leverages the prediction of conformational properties of proteins from AlphaFold2 and associates them with the propensity for co-condensation as reported from experimental measurements in the CD-CODE database.

## Materials and Methods

### Datasets of Protein Condensates for the Training and Testing of CoDropleT.

Protein condensate data on only naturally occurring human condensates were obtained from CD-CODE (48). CD-CODE was used to generate two sets of data for training CoDropleT: (i) PPS-positive protein pairs, and (ii) PPS-negative protein pairs. To train CoDropleT, we generated a set of PPS-positive pairs by selecting containing *n* proteins and then forming pairs from the proteins found within each condensate. For instance, if a condensate contains proteins A, B, and C, the pairs would be: (1) A and B, (2) A and C, and (3) B and C. To generate the PPS-negative protein pairs, we incorporated condensates containing up to *m* proteins. For every protein within these condensates, we formed pairs and classified them as negative if they did not coexist in any biomolecular condensates. We set a limit on the sequence length for each protein at 1,024 amino acids. We allocated 80% of the dataset for training purposes and the remaining 20% for testing. For the positive dataset, we selected *n* = 50, as the protein components in larger condensates tend to be less reliable. This number represents a trade-off between having enough data for effective model training and maintaining data reliability. In determining the value of *m*, we made similar considerations. We chose *m* = 12, as increasing it further resulted in a significant decline in test metrics (*SI Appendix*, Table S2). Consequently, the final dataset, with *n* = 50 and *m* = 12, comprised 2,440 positive and 3,760 negative human protein pairs.

In addressing the potential bias wherein all proteins in our initial dataset could exist in biomolecular condensates, we expanded the dataset by including additional protein pairs. These pairs were formed by themselves, serving as indicators of their likelihood to be part of biomolecular condensates. We selected all proteins already identified within biomolecular condensates, to represent the positive group. Building the negative dataset is a more complicated task, due to the lack of publicly available resources that report proteins that do not form condensates, as well as the fact that condensates may form only under specific conditions. We define our negative dataset based on the InWeb3 database protein–protein interaction network (49). All proteins that had direct connections with known condensate proteins were excluded, under the assumption that this would reduce the probability of false negatives. The final nonredundant datasets, filtered by 50% sequence identity, contained 1,791 PPS-positive and 1,579 PPS-negative human proteins.

### Training and Testing of CoDropleT.

The PPS-positive and PPS-negative protein pair datasets were split into training (80%) and testing datasets (20%). Resampling was applied during training to balance the number of positive and negative data points. The training step involved training the transformer network to recognize patterns in the Alphafold2-encoded protein representations that indicate phase separation behavior. At each step, we utilized a minibatch of size four. The output and loss were calculated using the network, with an L2 regularisation term added to the loss. The gradient of the loss with respect to the parameters of the network was calculated and used to update the parameters. To mitigate the risk of overfitting and to ensure that the model was generalizable to unseen data, we employed cross-validation to optimize the hyperparameters. To ensure a manageable training time, we imposed a limit on the maximum length of protein sequences to 1,024. Training took place over 40 epochs on a high-performance computing environment using two Graphics Processing Unit (GPU) cores. The model was tested using the hold-out dataset, which comprised 20% of our original data, both for PPS positive and PPS negative protein pairs. The performance of the model was evaluated based on its ability to correctly predict PPS behavior, using metrics such as precision, recall, and AUC for the receiver operating characteristic (ROC) curve.

### Model Algorithm and Pseudocode.

In the following sections, we describe the algorithm in detail using a pseudocode, with the following conventions: scalar values are represented in italics, while vectors are represented in bold (e.g., **n** represents the vector of nodes, and **e** represents the vector of edges). We use the indices *i* and *j* to denote the residue dimension. We use capitalized operator names when they encapsulate learned parameters. For functions without parameters, we use lowercase operator names. More details about the functions can be found in the following. We use ☉ for element-wise multiplication, ⊕ for the outer sum, and **a**^⊤^**b** for the dot product of vectors **a** and **b**.

#### Basic components and blocks.

The following basic functions are utilized in the subsequent pseudocode:

• Linear: Represents a linear transformation that involves multiplying the input by a weight matrix *W* and adding a bias vector **b**.
• LinearNoBias: Denotes a linear transformation without the bias vector **b**. It involves only the multiplication of the input by the weight matrix *W*.
• LayerNorm: This refers to layer normalization. It operates on the channel dimensions and includes learnable per-channel gains and biases.
• relu: This term denotes the rectified linear unit (ReLU) activation function. It is a nonlinear function that outputs the input directly if it is positive; otherwise, it outputs zero.
• sigmoid: This function denotes the sigmoid activation function that maps the input to a value in the range of 0 to 1, using the equation: $sigmoid\left(x\right)=1/\left(1+e^{-x}\right)$.
• ${softmax}_{\mathrm{ind}}$: This function signifies the softmax activation function that normalizes a vector of numerical values into a probability distribution over the indices *ind*.
• ${dropout}_{\mathrm{rate}}$: This term designates the rate parameter of the dropout regularization technique. This method randomly nullifies a certain proportion (*rate*) of input units during training as a preventive measure against overfitting.
• ${mean}_{\mathrm{ind}}$: This function represents a process for calculating the mean value. It computes the average value across a specified index, *ind*.
• ${concat}_{\mathrm{ind}}$: This function is used for concatenating vectors based on the specified index, *ind*. If *ind* is not provided, it defaults to concatenating two vectors into a single vector.

#### Transition block.

The transition block facilitates nonlinear transitions for the features of nodes or edges, while maintaining dimension invariance. The transition block comprises a layer normalization, applied to the input activation, followed by a standard 2-layer multilayer perceptron (MLP) which uses the ReLU activation function. The intermediate number of channels in the MLP expands the original number of channels by a factor of *n*_expand_ = 4. If we assume an input activation, denoted as a vector **a** ε ∈ $R^{c}$, where c represents the dimension of a, then the algorithm can be elucidated as follows:

***Algorithm 1:*** Transition block.

**Table:** 

def Transition ($a,n_{expand}=4$):		
1:	$a^{\prime }=\mathrm{Linear}\left(a\right)$	$a^{\prime }\in R^{c*n_{expand}}$
2:	**a** ← Linear(relu($a^{\prime }$))	
3:	**return a**

#### Self-attention block.

The self-attention block is responsible for updating a graph representation that consists of both node and edge features. It is a modified version of the basic self-attention with pair bias (45), designed to facilitate effective communication between the edge and node features. The attention block updates the edge features through the attention matrix, which is derived from node features. The number of attention heads *N_head_* is a hyperparameter. Assuming n*_i_* are features of dimension *c_n_*for each node, and e*_ij_* donates features for each pair of nodes, *i* and *j*, then the algorithm can be described as follows:

***Algorithm 2:*** Self-attention with pair bias.

**Table:** 

def SelfAttention $\left(\left\{n_{i}\right\},\left\{e_{\mathrm{ij}}\right\},N_{\text{head}}=8\right)$:	
*#* *Input projections*	
1:	$n_{i}\leftarrow$ LayerNorm ($n_{i}$)
2:	$e_{\mathrm{ij}}\leftarrow$LayerNorm($e_{\mathrm{ij}}$)
3:	$c_{n}^{\prime }=\frac{c_{n}}{N_{head}}$
4:	$q_{i}^{h},k_{i}^{h},v_{i}^{h},=\text{LinearNoBias}\left(n_{i}\right)$ $q_{i}^{h},k_{i}^{h},v_{i}^{h}\in R^{c},h\in \left\{1,\ldots ,N_{\text{head}}\right\}$
5:	$b_{\mathrm{ij}}^{h}=LinearNoBias\left(e_{\mathrm{ij}}\right)$
6:	$g_{i}^{h}=\mathrm{sigmoid}\left(\mathrm{Linear}\left(n_{i}\right)\right)\;\;$ $g_{i}^{h}\in R^{c_{n}^{\prime }}$
***#*** *Attention*	
7:	$a_{\mathrm{ij}}^{h}=\frac{1}{\sqrt{c}}q_{i}^{h⊤}k_{i}^{h}$
***#*** *Output edge features*	
8:	$e_{\mathrm{ij}}\leftarrow Linear\left(relu\left(Linear\left({concat}_{h}\left(a_{\mathrm{ij}}^{h}\right)\right)\right)\right)$
***#*** *Output node features*	
9:	$a_{\mathrm{ij}}^{h}\leftarrow {softmax}_{j}\left(a_{\mathrm{ij}}^{h}+b_{\mathrm{ij}}^{h}\right)$
10:	$o_{i}^{h}=g_{i}^{h}⊙\sum_{j}a_{\mathrm{ij}}^{h}v_{i}^{h}$
11:	$n_{i}\leftarrow \mathrm{Linear}\left({\mathrm{concat}}_{h}\left(o_{i}^{h}\right)\right)$
12:	**return** $\left\{n_{i}\right\},\;\left\{e_{\mathrm{ij}}\right\}$

#### Protein encoder.

The protein encoder takes the intermediate features from AlphaFold2, namely {**f**^msa^_i_},{**f**^pair^_ij_}, and {**f**^struc^_i_}, as input. These are transformed into initial representations for nodes {**n**^(0)^_i_} and representations for edges as pairs of residues {**e**^(0)^*_ij_*} in the model. Within this model, the node dimension (*c*_n_) is set to 256, while the edge dimension (*c*_e_) is set to 32. The representations of two proteins are encoded separately. These two representations are then concatenated to form a single one for the complex—residues are concatenated, and residue pairs between the two proteins are padded with zero vectors. A relative positional encoding is also added to the pair representation. This encoding is a binary value used to distinguish whether a residue pair comes from the same chain or a different chain.

***Algorithm 3:*** Encoding input protein representations from AlphaFold2.

**Table:** 

def ProteinEncoder$\left(\left\{f_{i}^{\mathrm{msa}}\right\},\left\{f_{\mathrm{ij}}^{\mathrm{pair}}\right\},\left\{f_{i}^{\mathrm{struc}}\right\}\right)$:		
*#* *Input projections*		
1:	$f_{i}^{\mathrm{msa}}\leftarrow$LayerNorm(Transition($f_{i}^{\mathrm{msa}}$))	
2:	$f_{\mathrm{ij}}^{\mathrm{pair}}\leftarrow$Transi LayerNorm (Transition ($f_{\mathrm{ij}}^{\mathrm{pair}}$))	
3:	$f_{i}^{\mathrm{struc}}\leftarrow$LayerNorm(Transition($f_{i}^{\mathrm{struc}}$))	
***#*** *Concatenate residue-wise features*		
4:	$f_{i}^{\mathrm{resi}}=\mathrm{concat}\left(f_{i}^{\mathrm{msa}},f_{i}^{\mathrm{struc}}\right)$	$f_{i}^{\mathrm{resi}}\in R^{c_{\mathrm{msa}}+c_{\mathrm{struct}}}$
***#*** *Output projection, one dimension of pair representations is reserved for relative positional encoding.*		
5:	$e_{\mathrm{ij}}^{\left(0\right)}\leftarrow \mathrm{Transition}\left(f_{\mathrm{ij}}^{\mathrm{pair}}\right)$	$e_{\mathrm{ij}}^{\left(0\right)}\in R^{c_{e}-1}$
6:	$n_{i}^{\left(0\right)}\leftarrow \mathrm{Transition}\left(f_{i}^{\mathrm{resi}}\right)$	$n_{i}^{\left(0\right)}\in R^{c_{n}}$
7:	**return** $\left\{n_{i}^{\left(0\right)}\right\},\;\left\{e_{\mathrm{ij}}^{\left(0\right)}\right\}$

#### Transformer blocks.

After the protein has been encoded, a self-attention block followed by a transition layer for nonlinear transformation allows interaction between edge and node information. The output of each layer is added to the current representations via a residual connection (50) and passed through a Dropout layer (51) before they are added together.

***Algorithm 4:*** Update node representations and edge representations.

**Table:** 

def TransformerBlock$\left(\left\{n_{i}^{\left(m\right)}\right\},\;\left\{e_{\mathrm{ij}}^{\left(m\right)}\right\}\right)$:
*#* *Communication and transition*
1:$\left\{n_{i}^{\left(m\right)}\right\},\left\{e_{\mathrm{ij}}^{\left(m\right)}\right\}+={\mathrm{dropout}}_{drop\_rate_{\mathrm{attention}}}(\mathrm{SelfAttention}\;\left(\left\{n_{i}^{\left(m\right)}\right\},\left\{e_{\mathrm{ij}}^{\left(m\right)}\right\}\right)$
2:$n_{i}^{\left(m+1\right)}={\mathrm{dropout}}_{drop\_rate_{\mathrm{transition}}}\left(\mathrm{Transition}\left(\mathrm{LayerNorm}\left(n_{i}^{\left(m\right)}\right)\right)\right)+n_{i}^{\left(m\right)}$
3:$e_{\mathrm{ij}}^{\left(m+1\right)}={\mathrm{dropout}}_{drop\_rate_{\mathrm{transition}}}\left(\mathrm{Transition}\left(\mathrm{LayerNorm}\left(e_{\mathrm{ij}}^{\left(m\right)}\right)\right)\right)+e_{\mathrm{ij}}^{\left(m\right)}$
4:**return**$\left\{n_{i}^{\left(m+1\right)}\right\},\;\left\{e_{\mathrm{ij}}^{\left(m+1\right)}\right\}$

#### PPS prediction head.

After passing through eight layers of cross-attention modules, we obtain the final representations of the protein residues. We apply average pooling to obtain a representation of the whole protein ${\hat{n}}^{protein}$, which is used for predicting PPS propensity. During the training phase, we use the cross-entropy loss function to calculate the loss in PPS prediction.

***Algorithm 5:*** Predict PPS propensity.

**Table:** 

def AffinityHead $\left(\left\{n_{i}\right\},\left\{n_{k}\right\},true\_affinity\right)$:		
*#* *Input projection*		
1:	$n_{i}\leftarrow \mathrm{Linear}\left(\mathrm{relu}\left(\mathrm{Linear}\left({\mathrm{dropout}}_{drop\_rate_{\mathrm{affinity}}}\left(\mathrm{LayerNorm}\left(n_{i}\right)\right)\right)\right)\right)$	
***#*** *Average pooling*		
2:	${\hat{n}}^{\mathrm{protein}}={\mathrm{mean}}_{i}\left(\left\{n_{i}\right\}\right)$	
***#*** *Output projection*		
6:	$PPS=\mathrm{sigmoid}(\mathrm{Linear}(\mathrm{relu}(\mathrm{Linear}(\mathrm{relu}(\mathrm{Linear}(\hat{n}))))))$	
***#*** *Calculate loss when training*		
7:	$L_{\mathrm{PPS}}=-true\_PPS*\log\left(\mathrm{PPS}\right)-\left(1-true\_PPS\right)*\log\left(1-PPS\right)$	
8:	**return** $PPS,\;L_{\mathrm{PPS}}$

#### AlphaFold2-generated protein representations.

We utilized pretrained AlphaFold2 to extract protein representations, which consist of three key components: i) a single sequence representation $\left\{f_{i}^{\mathrm{msa}}\right\}$, where $\;f_{i}^{\mathrm{msa}}\in R^{c_{\mathrm{msa}}}$, $c_{msa}=384$, and $\;i\in \;\left\{1\ldots \;N_{\mathrm{res}}\right\}$, derived by linearly projecting the first row of the MSA (multiple sequence alignment) representation. The MSA representation is the output of the final layer of the Evoformer and serves as the input to the structure module in AlphaFold2. ii) Pair representations $\left\{f_{i}^{\text{struc}}\right\}$ for each pair of residues *i* and *j*, where $f_{\mathrm{ij}}^{\mathrm{pair}}\in \;R^{c_{\mathrm{pair}}}$, $c_{pair}=128$. The pair representations $\left\{f_{\mathrm{ij}}^{\mathrm{pair}}\right\}$ is also output by the Evoformer and the input to the structure module in AlphaFold2, where it is used for predicting interresidue distances. iii) The structure representation $\left\{f_{i}^{\mathrm{struc}}\right\}$, obtained from the final layer of the structure module in AlphaFold2, where $f_{i}^{\mathrm{struc}}\in R^{c_{\mathrm{struc}}}$, $c_{\mathrm{struc}}=384$, and $i\in \{1\cdots N_{res}\}$. This structure representation is employed in AlphaFold2 for predicting side-chain dihedral angles and model confidence prediction. The MSA was generated using MMseqs2 (default setting) on BFD/Mgnify and Uniclust30 (2021 03). No structural template was fed during the prediction. Model 1.1.1 of AlphaFold2 (45) was used for inference, and no structural template was used.

### Comparing Co-condensation of MLO Proteins against Random Distributions.

Three well-characterized MLOs were selected for this analysis: SGs, P-bodies, and Nucleolus. Only pairs with proteins within the set of 4,135 proteins for which CoDropleT scores were calculated were included in this analysis. These three MLOs were selected by selecting commonly studied functional biomolecular condensates with >50 positive confidence scoring proteins within CD-CODE, which had not been included in the training and testing of the CoDropleT model. For each MLO, all possible protein pair combinations were generated from their list of protein components. This was used to get the distribution of cocondensing propensities predicted by CoDropleT. Each MLO distribution was compared against distributions of five random samples of the same size as the studied MLO.

### Comparing Pairwise CoDropleT Scores of SG Scaffolds.

We select a set of high-confidence SG proteins from CD-CODE, determined as SG proteins that have recorded experimental evidence of localization within SGs for which we have pair-wise predictions with G3BP1, TIA1, DDX3X, and C9orf72. We then compare the pairwise CoDropleT scores of each of the 4 scaffolds with the set of high-confidence SG components against their cocondensation propensities with non-SG components. To generate the set of pairwise CoDropleT scores with non-SG components, we consider all scaffold-protein pairs for which the nonscaffold protein is not reported at all to localize in SGs within the CD-CODE dataset.

### Characterizing the Co-condensation Propensity of Cytosolic Protein Pairs.

A list of 4,957 human cytosolic proteins were downloaded from The Human Protein Atlas (https://www.proteinatlas.org/humanproteome/subcellular/cytosol). By further restricting the sequence length to 1,024, we ended up with a list of 4,135 sequences. We then calculated all possible pairs from these sequences, amounting to 8,547,045 pairs.

## Supplementary Material

Appendix 01 (PDF)

Dataset S01 (XLSX)

Dataset S02 (XLSX)

## Data Availability

All study data are included in the article and/or supporting information.
