# Supplementary material for: AlphaFold2-based prediction of the co-condensation propensity of proteins
Source: Proc Natl Acad Sci U S A. 2024 Aug 12;121(34):e2315005121. doi: 10.1073/pnas.2315005121 (PMC11348322; doi:10.1073/pnas.2315005121)
Supplement: Supplementary file 1 — Appendix 01 (PDF) [file pnas.2315005121.sapp.pdf]

Supporting Information

Dataset S1. Predicted co-condensation propensities of cytosolic human protein pairs. Each row represents a unique pair of proteins, identified by their respective UniProt IDs, without any specified order. The table lists the predicted propensity of each protein pair to co-condensate as p(co-condensation), alongside the individual FuzDrop scores for each protein (1), which indicate their propensity to undergo phase separation individually.

Dataset S2. Protein-wise statistics of co-condensation propensities with other proteins. For each protein, identified by its UniProt ID, the table quantifies the number of other proteins predicted to co-condensate with it (positive_count for propensities >0.8, negative_count for propensities <0.2). It also provides the mean, standard deviation, minimum, and maximum of the predicted co-condensation propensities across all other proteins.

Table S1. Comparative amino acid composition of proteins with high and low numbers of predicted possible co-condensation partners. This table provides a statistical comparison of amino acid composition between proteins predicted to engage with a greater number of co-condensation partners versus those with fewer partners. The column ΔAA indicates the mean difference in composition between the two groups, while the standard error provides the variability measure of the amino acid proportions within the low and high partner count groups. The p-value is calculated from a t-test assessing the significance of the differences between these groups. Lastly, the Kolmogorov-Smirnov (KS) max difference denotes the greatest disparity in the cumulative distribution functions for the amino acid composition between the two sets of proteins.

amino acid	ΔAA	standard error	p-value	KS max difference
A	0.0044	0.0018	7.22E-04	8.83E-05
R	0.0043	0.0017	3.62E-04	2.44E-06
N	0.0017	0.0011	3.00E-02	3.18E-04
D	0.0085	0.0013	1.10E-20	3.90E-26
C	-0.0102	0.0010	5.40E-38	8.32E-29
Q	-0.0013	0.0013	1.69E-01	5.32E-03
E	0.0092	0.0020	8.69E-11	2.61E-28
G	-0.0028	0.0016	1.46E-02	4.78E-04
H	-0.0043	0.0009	1.62E-11	1.64E-07
I	0.0050	0.0013	1.05E-07	7.94E-12
L	-0.0127	0.0021	2.20E-17	3.72E-20
K	0.0184	0.0021	5.37E-34	1.63E-45
M	0.0014	0.0007	4.11E-03	9.30E-08
F	-0.0052	0.0011	3.61E-10	8.91E-14
P	-0.0038	0.0019	6.14E-03	5.32E-03
S	-0.0090	0.0017	1.76E-13	1.23E-14
T	-0.0027	0.0011	5.32E-04	1.53E-03
W	-0.0042	0.0006	3.41E-24	3.79E-18
Y	0.0000	0.0009	9.63E-01	1.41E-02
V	0.0033	0.0013	4.08E-04	5.21E-08

Table S2. Summary of evaluation metrics for each model tested in this study. The accuracy represents the proportion of correctly classified instances out of the total instances, reflecting the overall correctness. The precision measures the ratio of true positive predictions to the sum of true positives and false positives, indicating the correctness among the predicted positive instances. The recall, also known as sensitivity, quantifies the ratio of true positives to the sum of true positives and false negatives, capturing the ability to correctly identify all relevant instances. The F1 score is the harmonic mean of precision and recall, providing a balance between the two and offering a single metric for model comparison. The AUC (area under the curve) is derived from the receiver operating characteristic (ROC) curve and shows the ability of the model to distinguish between the classes, with a value of 1 indicating perfect discrimination and a value 0.5 indicating no discrimination. The MCC (Matthews correlation coefficient) considers true and false positives and negatives, offering a balanced measure of binary classification performance, with a value of +1 indicating perfect prediction, a value of 0 random prediction, and a value of -1 inverse prediction.

Models	Accuracy	Precision	Recall	F1 score	AUC	MCC
n = 50, m = 15	0.721	0.531	0.451	0.488	0.674	0.300
n = 50, m = 12	0.832	0.667	0.820	0.737	0.874	0.623
n = 50, m = 10	0.830	0.898	0.740	0.811	0.931	0.670
n = 50, m = 5	0.813	0.879	0.815	0.845	0.900	0.614
n = 20, m = 3	0.809	0.896	0.811	0.851	0.876	0.590

Supporting References

1. M. Hardenberg, A. Horvath, V. Ambrus, M. Fuxreiter, M. Vendruscolo, Widespread occurrence of the droplet state of proteins in the human proteome. *Proc. Natl. Acad. Sci. USA* **117**, 33254-33262 (2020).
